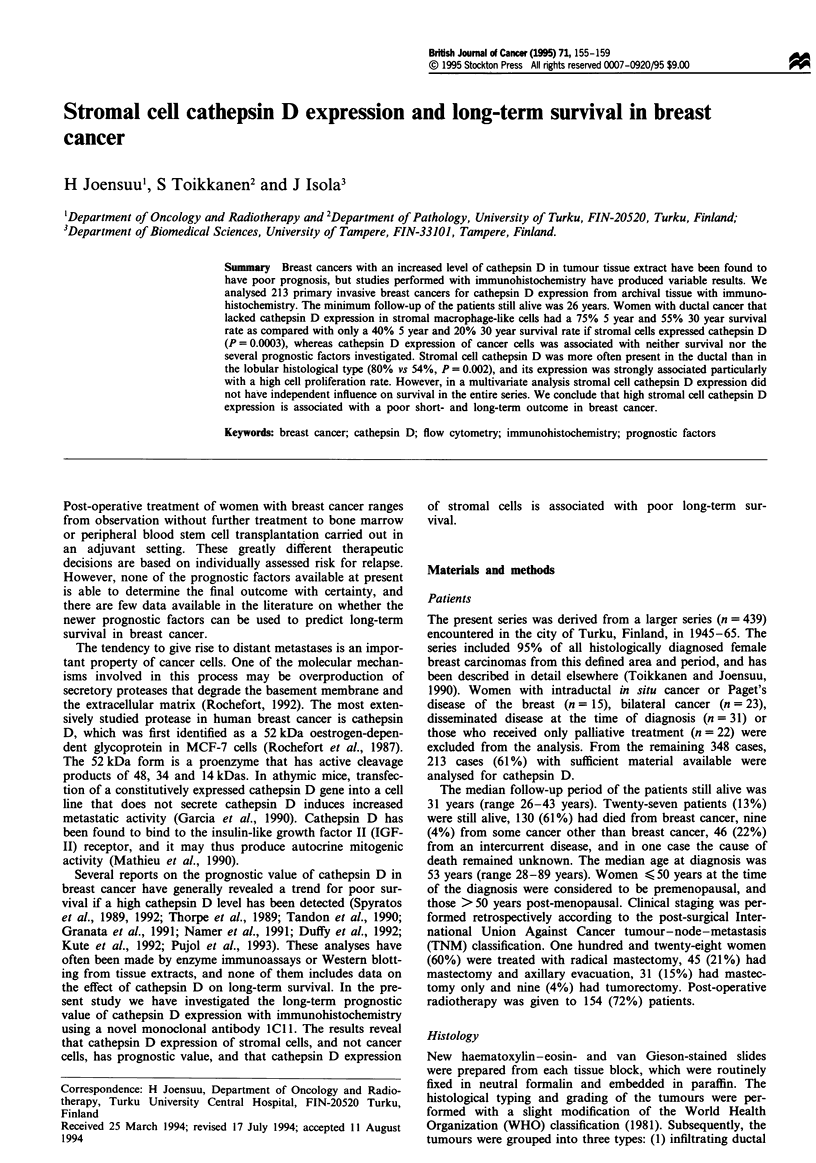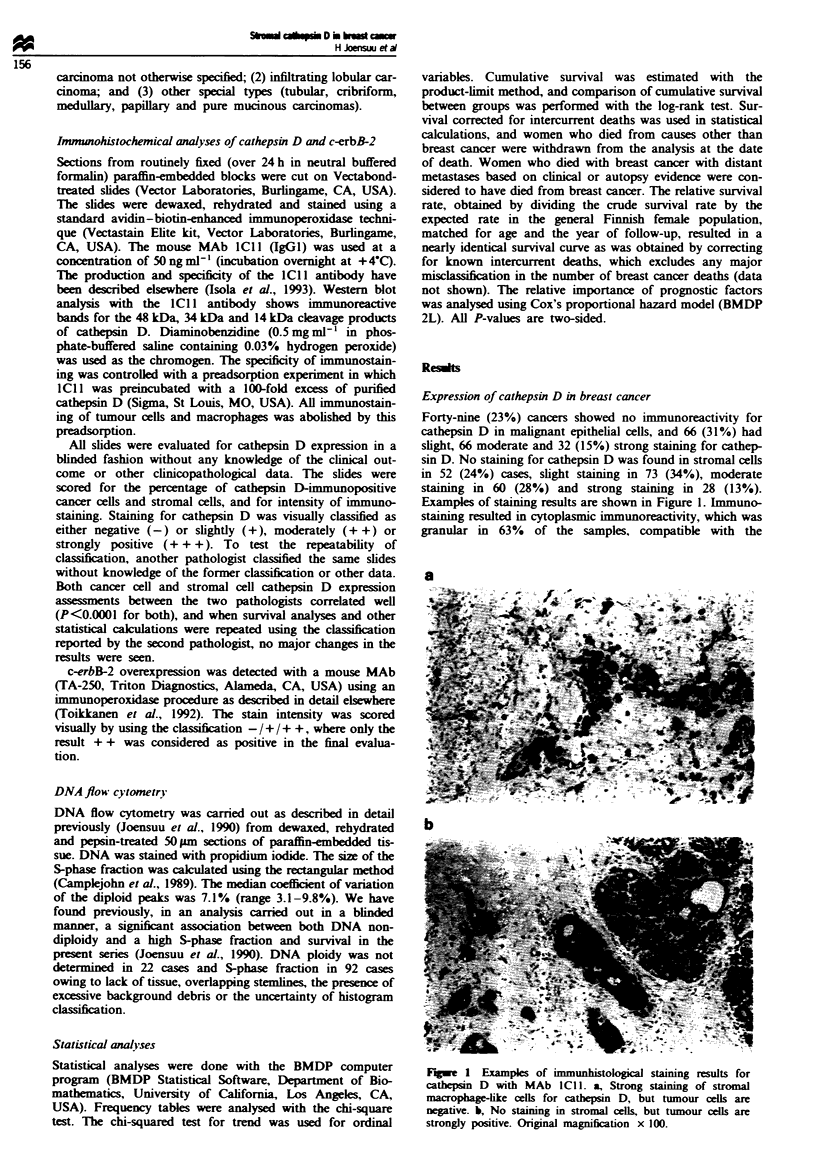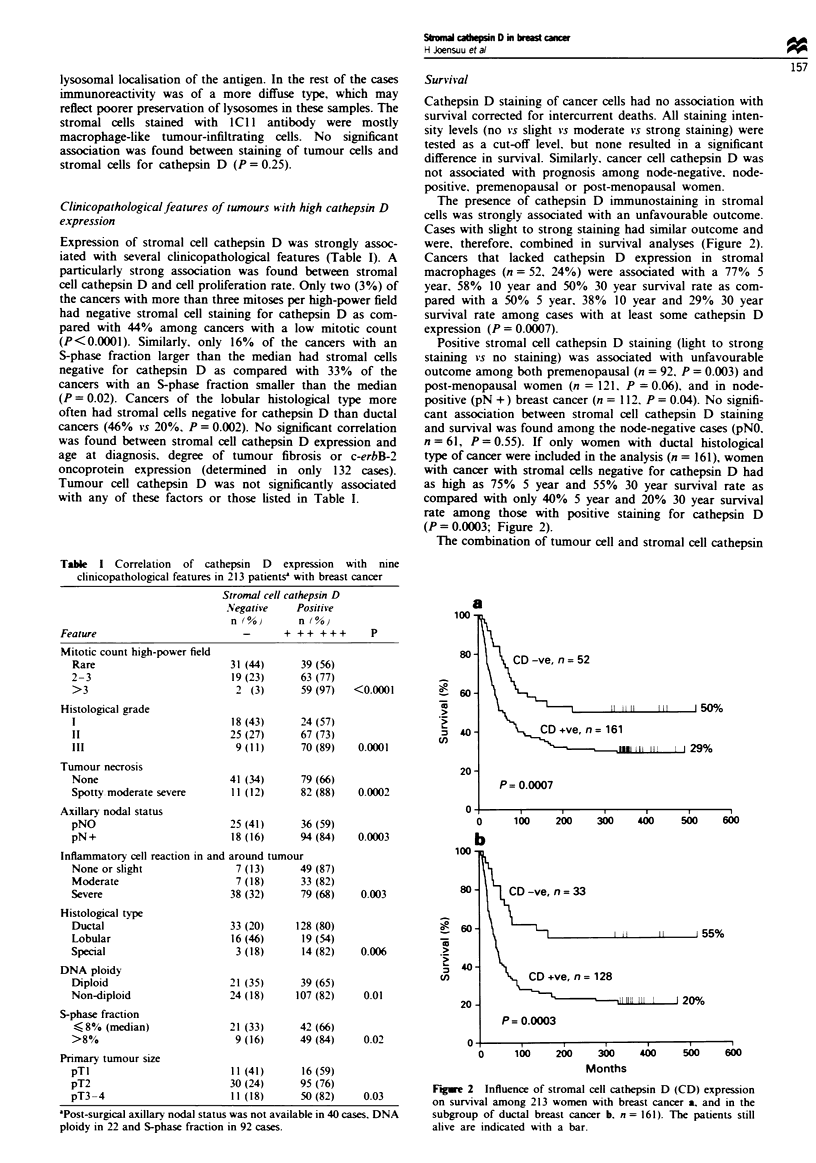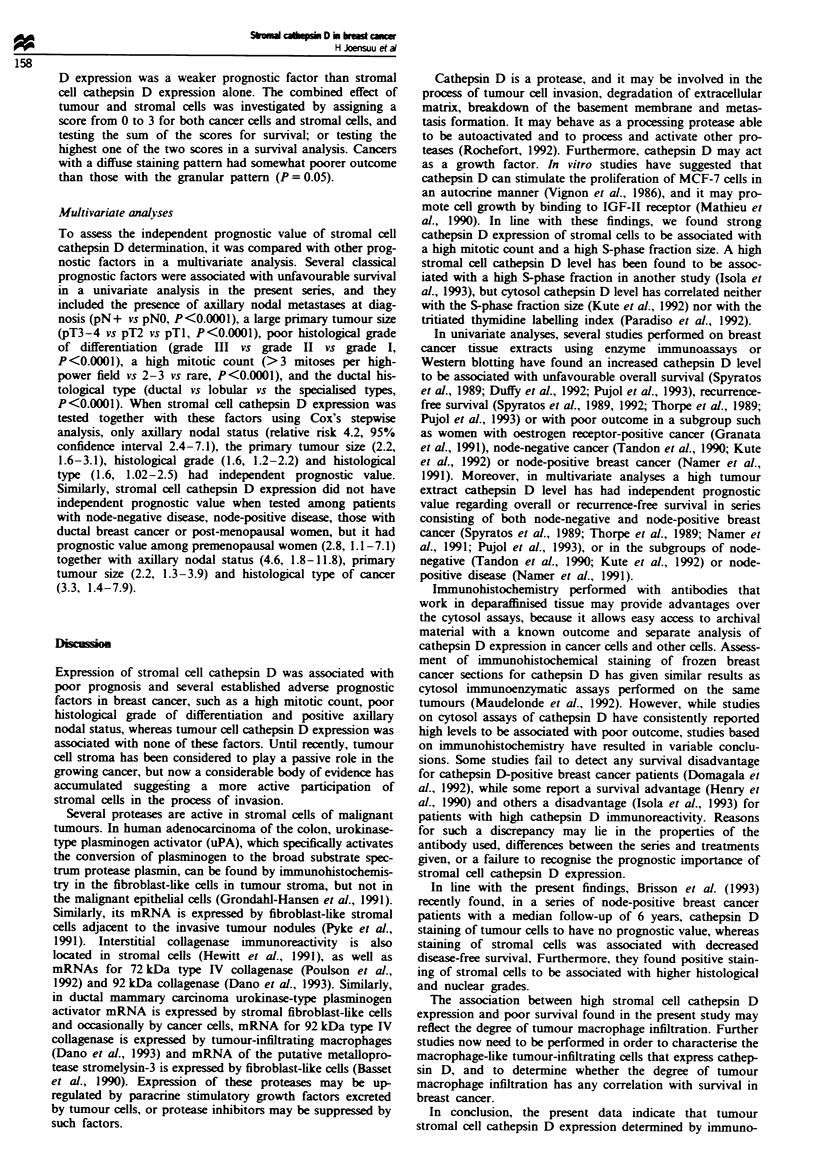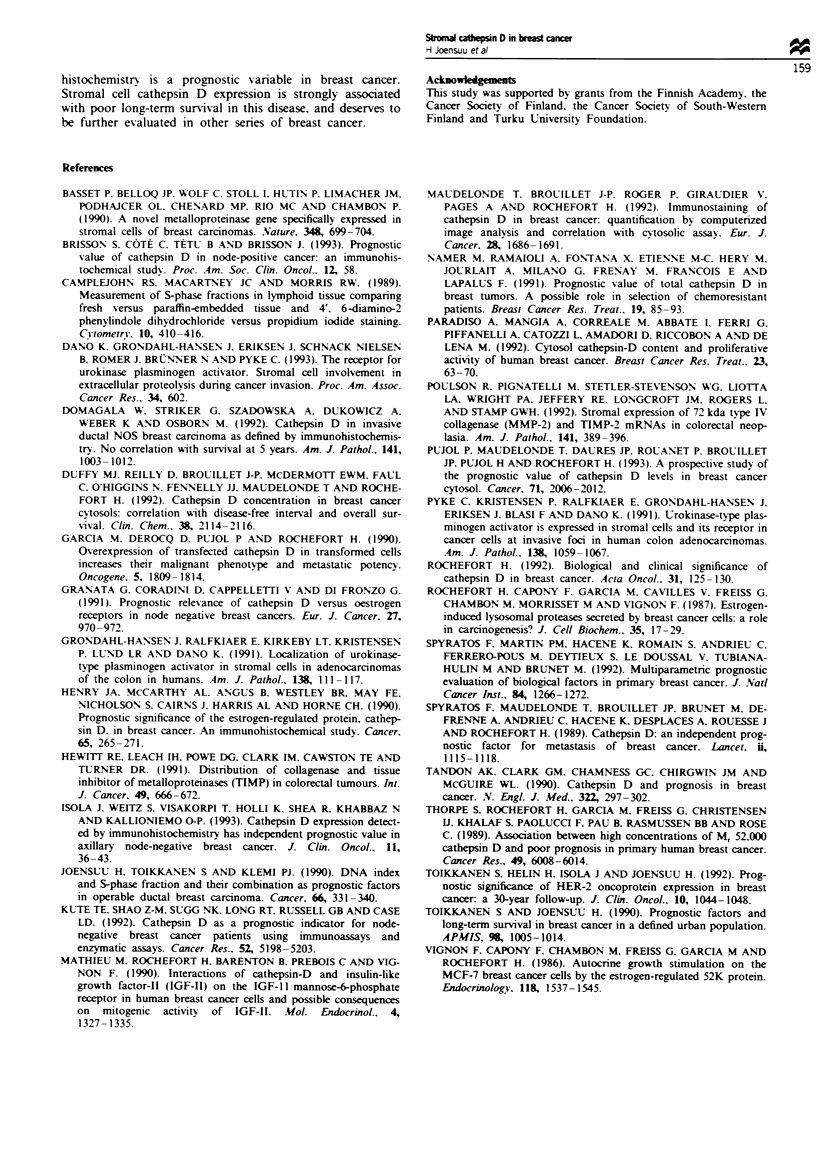# Stromal cell cathepsin D expression and long-term survival in breast cancer.

**DOI:** 10.1038/bjc.1995.32

**Published:** 1995-01

**Authors:** H. Joensuu, S. Toikkanen, J. Isola

**Affiliations:** Department of Oncology and Radiotherapy, University of Turku, Finland.

## Abstract

**Images:**


© 1995 Stockton Press All rights reserved 0007–0920/95 $9.00

### Stromal cell cathepsin D expression and long-term survival in breast cancer

H Joensuu[1], S Toikkanen[2] and J Isola[3]

[1]*Department of Oncology and Radiotherapy and* [2]*Department of Pathology, University of Turku, FIN-20520, Turku, Finland;* [3]*Department of Biomedical Sciences, University of Tampere, FIN-33101, Tampere, Finland.*

**Summary** Breast cancers with an increased level of cathepsin D in tumour tissue extract have been found to have poor prognosis, but studies performed with immunohistochemistry have produced variable results. We analysed 213 primary invasive breast cancers for cathepsin D expression from archival tissue with immunohistochemistry. The minimum follow-up of the patients still alive was 26 years. Women with ductal cancer that lacked cathepsin D expression in stromal macrophage-like cells had a 75% 5 year and 55% 30 year survival rate as compared with only a 40% 5 year and 20% 30 year survival rate if stromal cells expressed cathepsin D ($P = 0.0003$), whereas cathepsin D expression of cancer cells was associated with neither survival nor the several prognostic factors investigated. Stromal cell cathepsin D was more often present in the ductal than in the lobular histological type (80% *vs* 54%, $P = 0.002$), and its expression was strongly associated particularly with a high cell proliferation rate. However, in a multivariate analysis stromal cell cathepsin D expression did not have independent influence on survival in the entire series. We conclude that high stromal cell cathepsin D expression is associated with a poor short- and long-term outcome in breast cancer.

**Keywords:** breast cancer; cathepsin D; flow cytometry; immunohistochemistry; prognostic factors

Post-operative treatment of women with breast cancer ranges from observation without further treatment to bone marrow or peripheral blood stem cell transplantation carried out in an adjuvant setting. These greatly different therapeutic decisions are based on individually assessed risk for relapse. However, none of the prognostic factors available at present is able to determine the final outcome with certainty, and there are few data available in the literature on whether the newer prognostic factors can be used to predict long-term survival in breast cancer.

The tendency to give rise to distant metastases is an important property of cancer cells. One of the molecular mechanisms involved in this process may be overproduction of secretory proteases that degrade the basement membrane and the extracellular matrix (Rochefort, 1992). The most extensively studied protease in human breast cancer is cathepsin D, which was first identified as a 52 kDa oestrogen-dependent glycoprotein in MCF-7 cells (Rochefort *et al.*, 1987). The 52 kDa form is a proenzyme that has active cleavage products of 48, 34 and 14 kDas. In athymic mice, transfection of a constitutively expressed cathepsin D gene into a cell line that does not secrete cathepsin D induces increased metastatic activity (Garcia *et al.*, 1990). Cathepsin D has been found to bind to the insulin-like growth factor II (IGF-II) receptor, and it may thus produce autocrine mitogenic activity (Mathieu *et al.*, 1990).

Several reports on the prognostic value of cathepsin D in breast cancer have generally revealed a trend for poor survival if a high cathepsin D level has been detected (Spyratos *et al.*, 1989, 1992; Thorpe *et al.*, 1989; Tandon *et al.*, 1990; Granata *et al.*, 1991; Namer *et al.*, 1991; Duffy *et al.*, 1992; Kute *et al.*, 1992; Pujol *et al.*, 1993). These analyses have often been made by enzyme immunoassays or Western blotting from tissue extracts, and none of them includes data on the effect of cathepsin D on long-term survival. In the present study we have investigated the long-term prognostic value of cathepsin D expression with immunohistochemistry using a novel monoclonal antibody 1C11. The results reveal that cathepsin D expression of stromal cells, and not cancer cells, has prognostic value, and that cathepsin D expression of stromal cells is associated with poor long-term survival.

Correspondence: H Joensuu, Department of Oncology and Radiotherapy, Turku University Central Hospital, FIN-20520 Turku, Finland
Received 25 March 1994; revised 17 July 1994; accepted 11 August 1994

### Materials and methods

### *Patients*

The present series was derived from a larger series ($n = 439$) encountered in the city of Turku, Finland, in 1945–65. The series included 95% of all histologically diagnosed female breast carcinomas from this defined area and period, and has been described in detail elsewhere (Toikkanen and Joensuu, 1990). Women with intraductal *in situ* cancer or Paget's disease of the breast ($n = 15$), bilateral cancer ($n = 23$), disseminated disease at the time of diagnosis ($n = 31$) or those who received only palliative treatment ($n = 22$) were excluded from the analysis. From the remaining 348 cases, 213 cases (61%) with sufficient material available were analysed for cathepsin D.

The median follow-up period of the patients still alive was 31 years (range 26–43 years). Twenty-seven patients (13%) were still alive, 130 (61%) had died from breast cancer, nine (4%) from some cancer other than breast cancer, 46 (22%) from an intercurrent disease, and in one case the cause of death remained unknown. The median age at diagnosis was 53 years (range 28–89 years). Women $\leqslant 50$ years at the time of the diagnosis were considered to be premenopausal, and those $> 50$ years post-menopausal. Clinical staging was performed retrospectively according to the post-surgical International Union Against Cancer tumour–node–metastasis (TNM) classification. One hundred and twenty-eight women (60%) were treated with radical mastectomy, 45 (21%) had mastectomy and axillary evacuation, 31 (15%) had mastectomy only and nine (4%) had tumorectomy. Post-operative radiotherapy was given to 154 (72%) patients.

### *Histology*

New haematoxylin–eosin- and van Gieson-stained slides were prepared from each tissue block, which were routinely fixed in neutral formalin and embedded in paraffin. The histological typing and grading of the tumours were performed with a slight modification of the World Health Organization (WHO) classification (1981). Subsequently, the tumours were grouped into three types: (1) infiltrating ductal carcinoma not otherwise specified; (2) infiltrating lobular carcinoma; and (3) other special types (tubular, cribriform, medullary, papillary and pure mucinous carcinomas).

*Immunohistochemical analyses of cathepsin D and c-erbB-2*

Sections from routinely fixed (over 24 h in neutral buffered formalin) paraffin-embedded blocks were cut on Vectabond-treated slides (Vector Laboratories, Burlingame, CA, USA). The slides were dewaxed, rehydrated and stained using a standard avidin–biotin-enhanced immunoperoxidase technique (Vectastain Elite kit, Vector Laboratories, Burlingame, CA, USA). The mouse MAb 1C11 (IgG1) was used at a concentration of 50 ng $ml^{-1}$ (incubation overnight at +4°C). The production and specificity of the 1C11 antibody have been described elsewhere (Isola *et al*., 1993). Western blot analysis with the 1C11 antibody shows immunoreactive bands for the 48 kDa, 34 kDa and 14 kDa cleavage products of cathepsin D. Diaminobenzidine (0.5 mg $ml^{-1}$ in phosphate-buffered saline containing 0.03% hydrogen peroxide) was used as the chromogen. The specificity of immunostaining was controlled with a preadsorption experiment in which 1C11 was preincubated with a 100-fold excess of purified cathepsin D (Sigma, St Louis, MO, USA). All immunostaining of tumour cells and macrophages was abolished by this preadsorption.

All slides were evaluated for cathepsin D expression in a blinded fashion without any knowledge of the clinical outcome or other clinicopathological data. The slides were scored for the percentage of cathepsin D-immunopositive cancer cells and stromal cells, and for intensity of immunostaining. Staining for cathepsin D was visually classified as either negative (−) or slightly (+), moderately (++) or strongly positive (+++). To test the repeatability of classification, another pathologist classified the same slides without knowledge of the former classification or other data. Both cancer cell and stromal cell cathepsin D expression assessments between the two pathologists correlated well ($P<0.0001$ for both), and when survival analyses and other statistical calculations were repeated using the classification reported by the second pathologist, no major changes in the results were seen.

c-*erb*B-2 overexpression was detected with a mouse MAb (TA-250, Triton Diagnostics, Alameda, CA, USA) using an immunoperoxidase procedure as described in detail elsewhere (Toikkanen *et al*., 1992). The stain intensity was scored visually by using the classification −/+/++, where only the result ++ was considered as positive in the final evaluation.

*DNA flow cytometry*

DNA flow cytometry was carried out as described in detail previously (Joensuu *et al*., 1990) from dewaxed, rehydrated and pepsin-treated 50 μm sections of paraffin-embedded tissue. DNA was stained with propidium iodide. The size of the S-phase fraction was calculated using the rectangular method (Camplejohn *et al*., 1989). The median coefficient of variation of the diploid peaks was 7.1% (range 3.1–9.8%). We have found previously, in an analysis carried out in a blinded manner, a significant association between both DNA non-diploidy and a high S-phase fraction and survival in the present series (Joensuu *et al*., 1990). DNA ploidy was not determined in 22 cases and S-phase fraction in 92 cases owing to lack of tissue, overlapping stemlines, the presence of excessive background debris or the uncertainty of histogram classification.

*Statistical analyses*

Statistical analyses were done with the BMDP computer program (BMDP Statistical Software, Department of Biomathematics, University of California, Los Angeles, CA, USA). Frequency tables were analysed with the chi-square test. The chi-squared test for trend was used for ordinal variables. Cumulative survival was estimated with the product-limit method, and comparison of cumulative survival between groups was performed with the log-rank test. Survival corrected for intercurrent deaths was used in statistical calculations, and women who died from causes other than breast cancer were withdrawn from the analysis at the date of death. Women who died with breast cancer with distant metastases based on clinical or autopsy evidence were considered to have died from breast cancer. The relative survival rate, obtained by dividing the crude survival rate by the expected rate in the general Finnish female population, matched for age and the year of follow-up, resulted in a nearly identical survival curve as was obtained by correcting for known intercurrent deaths, which excludes any major misclassification in the number of breast cancer deaths (data not shown). The relative importance of prognostic factors was analysed using Cox's proportional hazard model (BMDP 2L). All *P*-values are two-sided.

### Results

*Expression of cathepsin D in breast cancer*

Forty-nine (23%) cancers showed no immunoreactivity for cathepsin D in malignant epithelial cells, and 66 (31%) had slight, 66 moderate and 32 (15%) strong staining for cathepsin D. No staining for cathepsin D was found in stromal cells in 52 (24%) cases, slight staining in 73 (34%), moderate staining in 60 (28%) and strong staining in 28 (13%). Examples of staining results are shown in Figure 1. Immunostaining resulted in cytoplasmic immunoreactivity, which was granular in 63% of the samples, compatible with the a b

**Figure 1** Examples of immunhistological staining results for cathepsin D with MAb 1C11. **a**, Strong staining of stromal macrophage-like cells for cathepsin D, but tumour cells are negative. **b**, No staining in stromal cells, but tumour cells are strongly positive. Original magnification ×100.

lysosomal localisation of the antigen. In the rest of the cases immunoreactivity was of a more diffuse type, which may reflect poorer preservation of lysosomes in these samples. The stromal cells stained with 1C11 antibody were mostly macrophage-like tumour-infiltrating cells. No significant association was found between staining of tumour cells and stromal cells for cathepsin D ($P = 0.25$).

### *Clinicopathological features of tumours with high cathepsin D expression*

Expression of stromal cell cathepsin D was strongly associated with several clinicopathological features (Table I). A particularly strong association was found between stromal cell cathepsin D and cell proliferation rate. Only two (3%) of the cancers with more than three mitoses per high-power field had negative stromal cell staining for cathepsin D as compared with 44% among cancers with a low mitotic count ($P < 0.0001$). Similarly, only 16% of the cancers with an S-phase fraction larger than the median had stromal cells negative for cathepsin D as compared with 33% of the cancers with an S-phase fraction smaller than the median ($P = 0.02$). Cancers of the lobular histological type more often had stromal cells negative for cathepsin D than ductal cancers (46% *vs* 20%, $P = 0.002$). No significant correlation was found between stromal cell cathepsin D expression and age at diagnosis, degree of tumour fibrosis or c-*erb*B-2 oncoprotein expression (determined in only 132 cases). Tumour cell cathepsin D was not significantly associated with any of these factors or those listed in Table I.

**Table I** Correlation of cathepsin D expression with nine clinicopathological features in 213 patients[a] with breast cancer

| | *Stromal cell cathepsin D* | | |
|---|---|---|---|
| | *Negative* n (%) | *Positive* n (%) | |
| *Feature* | – | + ++ +++ | P |
| Mitotic count high-power field | | | |
| Rare | 31 (44) | 39 (56) | |
| 2–3 | 19 (23) | 63 (77) | |
| >3 | 2 (3) | 59 (97) | <0.0001 |
| Histological grade | | | |
| I | 18 (43) | 24 (57) | |
| II | 25 (27) | 67 (73) | |
| III | 9 (11) | 70 (89) | 0.0001 |
| Tumour necrosis | | | |
| None | 41 (34) | 79 (66) | |
| Spotty moderate severe | 11 (12) | 82 (88) | 0.0002 |
| Axillary nodal status | | | |
| pNO | 25 (41) | 36 (59) | |
| pN+ | 18 (16) | 94 (84) | 0.0003 |
| Inflammatory cell reaction in and around tumour | | | |
| None or slight | 7 (13) | 49 (87) | |
| Moderate | 7 (18) | 33 (82) | |
| Severe | 38 (32) | 79 (68) | 0.003 |
| Histological type | | | |
| Ductal | 33 (20) | 128 (80) | |
| Lobular | 16 (46) | 19 (54) | |
| Special | 3 (18) | 14 (82) | 0.006 |
| DNA ploidy | | | |
| Diploid | 21 (35) | 39 (65) | |
| Non-diploid | 24 (18) | 107 (82) | 0.01 |
| S-phase fraction | | | |
| ≤8% (median) | 21 (33) | 42 (66) | |
| >8% | 9 (16) | 49 (84) | 0.02 |
| Primary tumour size | | | |
| pT1 | 11 (41) | 16 (59) | |
| pT2 | 30 (24) | 95 (76) | |
| pT3–4 | 11 (18) | 50 (82) | 0.03 |

[a]Post-surgical axillary nodal status was not available in 40 cases, DNA ploidy in 22 and S-phase fraction in 92 cases.

### *Survival*

Cathepsin D staining of cancer cells had no association with survival corrected for intercurrent deaths. All staining intensity levels (no *vs* slight *vs* moderate *vs* strong staining) were tested as a cut-off level, but none resulted in a significant difference in survival. Similarly, cancer cell cathepsin D was not associated with prognosis among node-negative, node-positive, premenopausal or post-menopausal women.

The presence of cathepsin D immunostaining in stromal cells was strongly associated with an unfavourable outcome. Cases with slight to strong staining had similar outcome and were, therefore, combined in survival analyses (Figure 2). Cancers that lacked cathepsin D expression in stromal macrophages ($n = 52$, 24%) were associated with a 77% 5 year, 58% 10 year and 50% 30 year survival rate as compared with a 50% 5 year, 38% 10 year and 29% 30 year survival rate among cases with at least some cathepsin D expression ($P = 0.0007$).

Positive stromal cell cathepsin D staining (light to strong staining *vs* no staining) was associated with unfavourable outcome among both premenopausal ($n = 92$, $P = 0.003$) and post-menopausal women ($n = 121$, $P = 0.06$), and in node-positive (pN +) breast cancer ($n = 112$, $P = 0.04$). No significant association between stromal cell cathepsin D staining and survival was found among the node-negative cases (pN0, $n = 61$, $P = 0.55$). If only women with ductal histological type of cancer were included in the analysis ($n = 161$), women with cancer with stromal cells negative for cathepsin D had as high as 75% 5 year and 55% 30 year survival rate as compared with only 40% 5 year and 20% 30 year survival rate among those with positive staining for cathepsin D ($P = 0.0003$; Figure 2).

The combination of tumour cell and stromal cell cathepsin

**Figure 2** Influence of stromal cell cathepsin D (CD) expression on survival among 213 women with breast cancer **a**, and in the subgroup of ductal breast cancer **b**, $n = 161$). The patients still alive are indicated with a bar.

D expression was a weaker prognostic factor than stromal cell cathepsin D expression alone. The combined effect of tumour and stromal cells was investigated by assigning a score from 0 to 3 for both cancer cells and stromal cells, and testing the sum of the scores for survival; or testing the highest one of the two scores in a survival analysis. Cancers with a diffuse staining pattern had somewhat poorer outcome than those with the granular pattern ($P = 0.05$).

*Multivariate analyses*

To assess the independent prognostic value of stromal cell cathepsin D determination, it was compared with other prognostic factors in a multivariate analysis. Several classical prognostic factors were associated with unfavourable survival in a univariate analysis in the present series, and they included the presence of axillary nodal metastases at diagnosis (pN+ *vs* pN0, $P<0.0001$), a large primary tumour size (pT3-4 *vs* pT2 *vs* pT1, $P<0.0001$), poor histological grade of differentiation (grade III *vs* grade II *vs* grade I, $P<0.0001$), a high mitotic count (>3 mitoses per high-power field *vs* 2-3 *vs* rare, $P<0.0001$), and the ductal histological type (ductal *vs* lobular *vs* the specialised types, $P<0.0001$). When stromal cell cathepsin D expression was tested together with these factors using Cox's stepwise analysis, only axillary nodal status (relative risk 4.2, 95% confidence interval 2.4-7.1), the primary tumour size (2.2, 1.6-3.1), histological grade (1.6, 1.2-2.2) and histological type (1.6, 1.02-2.5) had independent prognostic value. Similarly, stromal cell cathepsin D expression did not have independent prognostic value when tested among patients with node-negative disease, node-positive disease, those with ductal breast cancer or post-menopausal women, but it had prognostic value among premenopausal women (2.8, 1.1-7.1) together with axillary nodal status (4.6, 1.8-11.8), primary tumour size (2.2, 1.3-3.9) and histological type of cancer (3.3, 1.4-7.9).

### Discussion

Expression of stromal cell cathepsin D was associated with poor prognosis and several established adverse prognostic factors in breast cancer, such as a high mitotic count, poor histological grade of differentiation and positive axillary nodal status, whereas tumour cell cathepsin D expression was associated with none of these factors. Until recently, tumour cell stroma has been considered to play a passive role in the growing cancer, but now a considerable body of evidence has accumulated suggesting a more active participation of stromal cells in the process of invasion.

Several proteases are active in stromal cells of malignant tumours. In human adenocarcinoma of the colon, urokinase-type plasminogen activator (uPA), which specifically activates the conversion of plasminogen to the broad substrate spectrum protease plasmin, can be found by immunohistochemistry in the fibroblast-like cells in tumour stroma, but not in the malignant epithelial cells (Grondahl-Hansen *et al.*, 1991). Similarly, its mRNA is expressed by fibroblast-like stromal cells adjacent to the invasive tumour nodules (Pyke *et al.*, 1991). Interstitial collagenase immunoreactivity is also located in stromal cells (Hewitt *et al.*, 1991), as well as mRNAs for 72 kDa type IV collagenase (Poulson *et al.*, 1992) and 92 kDa collagenase (Dano *et al.*, 1993). Similarly, in ductal mammary carcinoma urokinase-type plasminogen activator mRNA is expressed by stromal fibroblast-like cells and occasionally by cancer cells, mRNA for 92 kDa type IV collagenase is expressed by tumour-infiltrating macrophages (Dano *et al.*, 1993) and mRNA of the putative metalloprotease stromelysin-3 is expressed by fibroblast-like cells (Basset *et al.*, 1990). Expression of these proteases may be upregulated by paracrine stimulatory growth factors excreted by tumour cells, or protease inhibitors may be suppressed by such factors.

Cathepsin D is a protease, and it may be involved in the process of tumour cell invasion, degradation of extracellular matrix, breakdown of the basement membrane and metastasis formation. It may behave as a processing protease able to be autoactivated and to process and activate other proteases (Rochefort, 1992). Furthermore, cathepsin D may act as a growth factor. *In vitro* studies have suggested that cathepsin D can stimulate the proliferation of MCF-7 cells in an autocrine manner (Vignon *et al.*, 1986), and it may promote cell growth by binding to IGF-II receptor (Mathieu *et al.*, 1990). In line with these findings, we found strong cathepsin D expression of stromal cells to be associated with a high mitotic count and a high S-phase fraction size. A high stromal cell cathepsin D level has been found to be associated with a high S-phase fraction in another study (Isola *et al.*, 1993), but cytosol cathepsin D level has correlated neither with the S-phase fraction size (Kute *et al.*, 1992) nor with the tritiated thymidine labelling index (Paradiso *et al.*, 1992).

In univariate analyses, several studies performed on breast cancer tissue extracts using enzyme immunoassays or Western blotting have found an increased cathepsin D level to be associated with unfavourable overall survival (Spyratos *et al.*, 1989; Duffy *et al.*, 1992; Pujol *et al.*, 1993), recurrence-free survival (Spyratos *et al.*, 1989, 1992; Thorpe *et al.*, 1989; Pujol *et al.*, 1993) or with poor outcome in a subgroup such as women with oestrogen receptor-positive cancer (Granata *et al.*, 1991), node-negative cancer (Tandon *et al.*, 1990; Kute *et al.*, 1992) or node-positive breast cancer (Namer *et al.*, 1991). Moreover, in multivariate analyses a high tumour extract cathepsin D level has had independent prognostic value regarding overall or recurrence-free survival in series consisting of both node-negative and node-positive breast cancer (Spyratos *et al.*, 1989; Thorpe *et al.*, 1989; Namer *et al.*, 1991; Pujol *et al.*, 1993), or in the subgroups of node-negative (Tandon *et al.*, 1990; Kute *et al.*, 1992) or node-positive disease (Namer *et al.*, 1991).

Immunohistochemistry performed with antibodies that work in deparaffinised tissue may provide advantages over the cytosol assays, because it allows easy access to archival material with a known outcome and separate analysis of cathepsin D expression in cancer cells and other cells. Assessment of immunohistochemical staining of frozen breast cancer sections for cathepsin D has given similar results as cytosol immunoenzymatic assays performed on the same tumours (Maudelonde *et al.*, 1992). However, while studies on cytosol assays of cathepsin D have consistently reported high levels to be associated with poor outcome, studies based on immunohistochemistry have resulted in variable conclusions. Some studies fail to detect any survival disadvantage for cathepsin D-positive breast cancer patients (Domagala *et al.*, 1992), while some report a survival advantage (Henry *et al.*, 1990) and others a disadvantage (Isola *et al.*, 1993) for patients with high cathepsin D immunoreactivity. Reasons for such a discrepancy may lie in the properties of the antibody used, differences between the series and treatments given, or a failure to recognise the prognostic importance of stromal cell cathepsin D expression.

In line with the present findings, Brisson *et al.* (1993) recently found, in a series of node-positive breast cancer patients with a median follow-up of 6 years, cathepsin D staining of tumour cells to have no prognostic value, whereas staining of stromal cells was associated with decreased disease-free survival, Furthermore, they found positive staining of stromal cells to be associated with higher histological and nuclear grades.

The association between high stromal cell cathepsin D expression and poor survival found in the present study may reflect the degree of tumour macrophage infiltration. Further studies now need to be performed in order to characterise the macrophage-like tumour-infiltrating cells that express cathepsin D, and to determine whether the degree of tumour macrophage infiltration has any correlation with survival in breast cancer.

In conclusion, the present data indicate that tumour stromal cell cathepsin D expression determined by immunohistochemistry is a prognostic variable in breast cancer. Stromal cell cathepsin D expression is strongly associated with poor long-term survival in this disease, and deserves to be further evaluated in other series of breast cancer.

**Acknowledgements**

This study was supported by grants from the Finnish Academy, the Cancer Society of Finland, the Cancer Society of South-Western Finland and Turku University Foundation.

**References**

BASSET P, BELLOQ JP, WOLF C, STOLL I, HUTIN P, LIMACHER JM, PODHAJCER OL, CHENARD MP, RIO MC AND CHAMBON P. (1990). A novel metalloproteinase gene specifically expressed in stromal cells of breast carcinomas. *Nature*, **348,** 699-704.

BRISSON S, CÔTÉ C, TÊTU B AND BRISSON J. (1993). Prognostic value of cathepsin D in node-positive cancer: an immunohistochemical study. *Proc. Am. Soc. Clin. Oncol.*, **12,** 58.

CAMPLEJOHN RS, MACARTNEY JC AND MORRIS RW. (1989). Measurement of S-phase fractions in lymphoid tissue comparing fresh versus paraffin-embedded tissue and 4', 6-diamino-2 phenylindole dihydrochloride versus propidium iodide staining. *Cytometry*, **10,** 410-416.

DANO K, GRONDAHL-HANSEN J, ERIKSEN J, SCHNACK NIELSEN B, ROMER J, BRÜNNER N AND PYKE C. (1993). The receptor for urokinase plasminogen activator. Stromal cell involvement in extracellular proteolysis during cancer invasion. *Proc. Am. Assoc. Cancer Res.*, **34,** 602.

DOMAGALA W, STRIKER G, SZADOWSKA A, DUKOWICZ A, WEBER K AND OSBORN M. (1992). Cathepsin D in invasive ductal NOS breast carcinoma as defined by immunohistochemistry. No correlation with survival at 5 years. *Am. J. Pathol.*, **141,** 1003-1012.

DUFFY MJ, REILLY D, BROUILLET J-P, McDERMOTT EWM, FAUL C, O'HIGGINS N, FENNELLY JJ, MAUDELONDE T AND ROCHEFORT H. (1992). Cathepsin D concentration in breast cancer cytosols: correlation with disease-free interval and overall survival. *Clin. Chem.*, **38,** 2114-2116.

GARCIA M, DEROCQ D, PUJOL P AND ROCHEFORT H. (1990). Overexpression of transfected cathepsin D in transformed cells increases their malignant phenotype and metastatic potency. *Oncogene*, **5,** 1809-1814.

GRANATA G, CORADINI D, CAPPELLETTI V AND DI FRONZO G. (1991). Prognostic relevance of cathepsin D versus oestrogen receptors in node negative breast cancers. *Eur. J. Cancer*, **27,** 970-972.

GRONDAHL-HANSEN J, RALFKIAER E, KIRKEBY LT, KRISTENSEN P, LUND LR AND DANO K. (1991). Localization of urokinase-type plasminogen activator in stromal cells in adenocarcinomas of the colon in humans. *Am. J. Pathol.*, **138,** 111-117.

HENRY JA, McCARTHY AL, ANGUS B, WESTLEY BR, MAY FE, NICHOLSON S, CAIRNS J, HARRIS AL AND HORNE CH. (1990). Prognostic significance of the estrogen-regulated protein, cathepsin D, in breast cancer. An immunohistochemical study. *Cancer*, **65,** 265-271.

HEWITT RE, LEACH IH, POWE DG, CLARK IM, CAWSTON TE AND TURNER DR. (1991). Distribution of collagenase and tissue inhibitor of metalloproteinases (TIMP) in colorectal tumours. *Int. J. Cancer*, **49,** 666-672.

ISOLA J, WEITZ S, VISAKORPI T, HOLLI K, SHEA R, KHABBAZ N AND KALLIONIEMO O-P. (1993). Cathepsin D expression detected by immunohistochemistry has independent prognostic value in axillary node-negative breast cancer. *J. Clin. Oncol.*, **11,** 36-43.

JOENSUU H, TOIKKANEN S AND KLEMI PJ. (1990). DNA index and S-phase fraction and their combination as prognostic factors in operable ductal breast carcinoma. *Cancer*, **66,** 331-340.

KUTE TE, SHAO Z-M, SUGG NK, LONG RT, RUSSELL GB AND CASE LD. (1992). Cathepsin D as a prognostic indicator for node-negative breast cancer patients using immunoassays and enzymatic assays. *Cancer Res.*, **52,** 5198-5203.

MATHIEU M, ROCHEFORT H, BARENTON B, PREBOIS C AND VIGNON F. (1990). Interactions of cathepsin-D and insulin-like growth factor-II (IGF-II) on the IGF-II mannose-6-phosphate receptor in human breast cancer cells and possible consequences on mitogenic activity of IGF-II. *Mol. Endocrinol.*, **4,** 1327-1335.

MAUDELONDE T, BROUILLET J-P, ROGER P, GIRAUDIER V, PAGES A AND ROCHEFORT H. (1992). Immunostaining of cathepsin D in breast cancer: quantification by computerized image analysis and correlation with cytosolic assay. *Eur. J. Cancer*, **28,** 1686-1691.

NAMER M, RAMAIOLI A, FONTANA X, ETIENNE M-C, HERY M, JOURLAIT A, MILANO G, FRENAY M, FRANCOIS E AND LAPALUS F. (1991). Prognostic value of total cathepsin D in breast tumors. A possible role in selection of chemoresistant patients. *Breast Cancer Res. Treat.*, **19,** 85-93.

PARADISO A, MANGIA A, CORREALE M, ABBATE I, FERRI G, PIFFANELLI A, CATOZZI L, AMADORI D, RICCOBON A AND DE LENA M. (1992). Cytosol cathepsin-D content and proliferative activity of human breast cancer. *Breast Cancer Res. Treat.*, **23,** 63-70.

POULSON R, PIGNATELLI M, STETLER-STEVENSON WG, LIOTTA LA, WRIGHT PA, JEFFERY RE, LONGCROFT JM, ROGERS L AND STAMP GWH. (1992). Stromal expression of 72 kda type IV collagenase (MMP-2) and TIMP-2 mRNAs in colorectal neoplasia. *Am. J. Pathol.*, **141,** 389-396.

PUJOL P, MAUDELONDE T, DAURES JP, ROUANET P, BROUILLET JP, PUJOL H AND ROCHEFORT H. (1993). A prospective study of the prognostic value of cathepsin D levels in breast cancer cytosol. *Cancer*, **71,** 2006-2012.

PYKE C, KRISTENSEN P, RALFKIAER E, GRONDAHL-HANSEN J, ERIKSEN J, BLASI F AND DANO K. (1991). Urokinase-type plasminogen activator is expressed in stromal cells and its receptor in cancer cells at invasive foci in human colon adenocarcinomas. *Am. J. Pathol.*, **138,** 1059-1067.

ROCHEFORT H. (1992). Biological and clinical significance of cathepsin D in breast cancer. *Acta Oncol.*, **31,** 125-130.

ROCHEFORT H, CAPONY F, GARCIA M, CAVILLES V, FREISS G, CHAMBON M, MORRISSET M AND VIGNON F. (1987). Estrogen-induced lysosomal proteases secreted by breast cancer cells: a role in carcinogenesis? *J. Cell Biochem.*, **35,** 17-29.

SPYRATOS F, MARTIN PM, HACENE K, ROMAIN S, ANDRIEU C, FERRERO-POUS M, DEYTIEUX S, LE DOUSSAL V, TUBIANA-HULIN M AND BRUNET M. (1992). Multiparametric prognostic evaluation of biological factors in primary breast cancer. *J. Natl Cancer Inst.*, **84,** 1266-1272.

SPYRATOS F, MAUDELONDE T, BROUILLET JP, BRUNET M, DEFRENNE A, ANDRIEU C, HACENE K, DESPLACES A, ROUESSE J AND ROCHEFORT H. (1989). Cathepsin D: an independent prognostic factor for metastasis of breast cancer. *Lancet*, **ii,** 1115-1118.

TANDON AK, CLARK GM, CHAMNESS GC, CHIRGWIN JM AND McGUIRE WL. (1990). Cathepsin D and prognosis in breast cancer. *N. Engl. J. Med.*, **322,** 297-302.

THORPE S, ROCHEFORT H, GARCIA M, FREISS G, CHRISTENSEN IJ, KHALAF S, PAOLUCCI F, PAU B, RASMUSSEN BB AND ROSE C. (1989). Association between high concentrations of $M_r$ 52,000 cathepsin D and poor prognosis in primary human breast cancer. *Cancer Res.*, **49,** 6008-6014.

TOIKKANEN S, HELIN H, ISOLA J AND JOENSUU H. (1992). Prognostic significance of HER-2 oncoprotein expression in breast cancer: a 30-year follow-up. *J. Clin. Oncol.*, **10,** 1044-1048.

TOIKKANEN S AND JOENSUU H. (1990). Prognostic factors and long-term survival in breast cancer in a defined urban population. *APMIS*, **98,** 1005-1014.

VIGNON F, CAPONY F, CHAMBON M, FREISS G, GARCIA M AND ROCHEFORT H. (1986). Autocrine growth stimulation on the MCF-7 breast cancer cells by the estrogen-regulated 52K protein. *Endocrinology*, **118,** 1537-1545.